# BREATHE: The Health Data Research Hub for Respiratory Health.

**DOI:** 10.23889/ijpds.v7i3.1994

**Published:** 2022-08-25

**Authors:** Chris Orton, David Ford, Aziz Sheikh, Jennifer Quint, Martin Tobin, Ian Hall, Christopher Griffiths, Jonathon Crompton, Monica Fletcher

**Affiliations:** 1 Swansea University; 2 University of Edinburgh; 3 Imperial College London; 4 University of Leicester; 5 University of Nottingham; 6 Queen Mary University of London

## Objectives

The BREATHE Health Data Research Hub for Respiratory Health was formed in October 2019 as a multi-site academic consortium with multiple industrial partners via an Industry Forum and across its wider network. BREATHE’s main mission is enhancing data services within respiratory science, funded by the UKRI Industrial Strategy Challenge Fund.

## Approach

BREATHE leveraged expertise across its founding sites and industrial partners to create data services which could be used by multiple sectors of collaborator. Across the founding sites, BREATHE was able to mobilise datasets housed within national TREs to form real-world evidence eCohorts for rapid and efficient respiratory study (Asthma, COPD, ILD), and has worked with specialists in cohort study and genomic data to house and supply these from within our partner TRE, SAIL Databank. As well as data assets, BREATHE is able to provide clinical and data expertise to collaborators for grant submissions and on bespoke respiratory science projects.

## Results

Including a significant period of work during the pandemic supporting COVID-19 research and also focusing on other respiratory disease science support, BREATHE is now well-placed to move towards a sustainable operating plan post-grant from March 2023. Due to the approach taken in maximising data services for multiple sectors, BREATHE is positioned to provide data linkage and sharing services (making use of its TRE, SAIL Databank), providing analytic and clinical support to respiratory research projects for customers in multiple sectors (Pharma, SMEs, Academia, NHS, Charities), and advancing synthetic data and software development, again in partnership with SAIL and our wider industry partners.

## Conclusion

As of March 2022, BREATHE has established a portfolio of data services and projects interfacing with multiple sectors of collaborator in enhancing respiratory science projects across the UK. With population-level data assets representing Wales, Scotland, and England and the ability to work with the Northern Ireland infrastructure housed at Swansea University, BREATHE supports 4-nation respiratory science in RWE data, and provides clinical and data linkage expertise to studies such as longitudinal cohorts, pharma companies, and contract research organisations.

